# Enhancement of Cell Recovery for Dissociated Human Embryonic Stem Cells After Cryopreservation

**DOI:** 10.1002/btpr.358

**Published:** 2009-12-11

**Authors:** Xia Xu, Sally Cowley, Christopher J Flaim, William James, Lenard W Seymour, Zhanfeng Cui

**Affiliations:** Dept. of Engineering Science, Institute of Biomedical Engineering, University of OxfordOxford, UK; Sir William Dunn School of Pathology, University of OxfordOxford, UK; Dept. of Clinical Pharmacology, University of OxfordOxford, UK

**Keywords:** Cryopreservation of human embryonic stem cells, cell recovery rate, ROCK inhibitor, p53 inhibitor

## Abstract

Due to widespread applications of human embryonic stem (hES) cells, it is essential to establish effective protocols for cryopreservation and subsequent culture of hES cells to improve cell recovery. We have developed a new protocol for cryopreservation of dissociated hES cells and subsequent culture. We examined the effects of new formula of freezing solution containing 7.5% dimethylsulfoxide (DMSO) (v/v %) and 2.5% polyethylene glycol (PEG) (w/v %) on cell survival and recovery of hES cells after cryopreservation, and further investigated the role of the combination of Rho-associated kinase (ROCK) inhibitor and p53 inhibitor on cell recovery during the subsequent culture. Compared with the conventional slow-freezing method which uses 10% DMSO as a freezing solution and then cultured in the presence of ROCK inhibitor at the first day of culture, we found out that hES cell recovery was significantly enhanced by around 30 % (*P* < 0.05) by the new freezing solution. Moreover, at the first day of post-thaw culture, the presence of 10 μM ROCK inhibitor (Y-27632) and 1 μM pifithrin-μ together further significantly improved cell recovery by around 20% (*P* < 0.05) either for feeder-dependent or feeder-independent culture. hES cells remained their undifferentiated status after using this novel protocol for cryopreservation and subsequent culture. Furthermore, this protocol is a scalable cryopreservation method for handling large quantities of hES cells. © 2009 American Institute of Chemical Engineers Biotechnol. Prog., 2010

## Introduction

Due to their potentially unlimited capacity for self-renewal and unique developmental potential to differentiate into all somatic cell types of the human body, hES cells have opened a new door for drug discovery, regenerative medicine, and tissue replacement after injury or disease.^1^, ^2^ However, an essential prerequisite for successful applications of hES cells is to develop efficient cryopreservation methods to overcome the current low cell recovery rate after cryopreservation.

Two freezing protocols are currently applied to cryopreserve hES cells, including slow-freezing and vitrification. Slow-freezing using 10% DMSO as a cryoprotectant is commonly used successfully to cryopreserve primary cells,^3^ human mesenchymal stem cells,^4^ and mouse embryonic stem cells.^5^ However, this protocol has not been successfully transferred to hES cells. It leads to poor cell survival rate after freezing.^6^, ^7^ On the other hand, vitrification of hES cells by the open pulled-straw method is much more effective than the slow freezing. A higher cell survival rate, 70–90%, is reported after vitrification.^8^, ^9^, ^10^ However, the fast-freezing protocols are much dependent on the researcher's experience and are too labour-intensive to be suitable for handling large quantities of hES cells during cryopreservation. Hence, it is crucial to develop an efficient and scalable cryopreservation method for the widespread applications of hES cells.

It is thought that low cell recovery rate after freezing is caused by apoptosis, which leads to cellular detachment and dissociation, rather than by cellular necrosis induced directly by freezing.^11^ The application of ROCK inhibitor, Y-27632, is reported to significantly diminish dissociation-induced apoptosis, and thereby increases the cell recovery rate after dissociation.^12^ Furthermore, it has been demonstrated that the presence of ROCK inhibitor during freezing and post-thawing can enhance the cell survival rate and colony formation.^13^, ^14^ In addition, reduction in p53 expression could reduce DNA-induced apoptosis.^15^

PEG, a neutral, water soluble, nontoxic polymer, plays an important role in protection of cells and organs against damage caused by cold storage.^16^ Cold storage can cause oxidative stress and further affect cell viability and cell recovery in injury.^14^ The production of reactive oxygen species (ROS) under oxidative stress can contribute to the modification of mitochondrial permeability.^17^ This can result in the release of cytochrome C (cyt c) from mitochondrial intermembranes into the cytoplasm, which then further activates caspase-9 activity, leading to apoptosis. The presence of PEG in the preservation solution could inhibit ROS production.^18^ Moreover, it can protect or repair the integrity of the glycocalyx (Gcx) which results in restoring its regulatory functions.^19^

Based on the previous research results, we have developed a novel protocol for cryopreservation of hES cells and subsequent culture. We describe an efficient cryopreservation method for slow-freezing of dissociated hES cells, which allows higher cell recovery rate and maintains undifferentiated status after cryopreservation.

## Materials and Methods

### Maintenance culture of hes cells

The human embryonic stem cell line, HUES2 (Howard Hughes Medical Institute, Department of Molecular and Cellular Biology, Harvard University, USA) which was approved by the UK Stem Cell Bank Steering Committee, was used to evaluate the efficiency of the protocol developed. Maintenance culture of hES cells was carried out by two different culture methods: feeder-dependent culture and feeder- independent culture. For the feeder-dependent culture, hES cells were cultured on a feeder layer of mitomycin C-inactivated mouse embryo fibroblast (MEF) in 0.1% gelatine-coated plate, in hES culture medium containing knockout Dulbecco's modified Eagle's medium, supplemented with 10% KO-Serum Replacement, 1% nonessential amino acids, 2 mM Glutamax-I (all from Invitrogen GIBCO, UK), 0.055 mM β-mercaptoethanol (GIBCO, UK), and 10 ng/mL basic fibroblast growth factor (bFGF) (R&D systems, UK) at 37°C, under 5% CO_2_, and 21% O_2_. Culture medium was changed 50% daily. For feeder-independent culture, the hES cells were cultured on matrigel-coated plates diluted 1:100, in MTeSR™ culture medium (Stem Cell Technologies, France), at 37°C, under 5% CO_2_ and 21% O_2_. Culture medium was completely changed daily.

For passaging, hES colonies were detached by TrypLE™ Express (Invitrogen GIBCO, UK) at 37°C for 5–7 min after 5–7 days of culture, followed by gentle flushing with pipette several times to detach hES cells. Undifferentiated dissociated hES cells were transferred to fresh MEFs plates or matrigel-coated plates which were prepared in advance, in the presence of 10 μM ROCK inhibitor Y-27632 (Merck Chemicals, UK) during the first day of culture. The split ratio was 1:5–1:10.

### Cryopreservation of hES cells

Due to the two different culture systems used in this study, two series of freezing solutions were prepared based on the culture system used. The details of composition of freezing solution is listed in Table 1. DMSO, 1, 2-propanediol, PEG with molecular weight of 20,000 (Sigma-aldrich, UK) were used in this study. The dissociated hES cells were mixed with freezing solution at an equal volume ratio, with the final cell density 1 x 10^6^ cells/cryovial and final volume of 0.5 ml at 4°C for 30 min, and then placed in a cryo-container (“Mr. Frosty”, Nalgene, Denmark), kept at −80°C overnight, then stored in liquid nitrogen for 1–2 weeks. Before use, the cells were rapidly thawed in water bath at 37°C. Immediately after thawing, the cell suspension was diluted to 10 mL PBS, and the added cryoprotectant agents (CPAs) were removed. The cells were resuspended in the culture medium at the required medium.

**Table 1:** Compositions of Freezing Solution

Freezing Solution	Concentration of DMSO (v/v %)	Concentration of PROH (v/v %)	PEG (wt/v %)	Y-27632 (μM)
10% DMSO	20			
10 % DMSO + 10μM ROCK inhibitor	20			20
7.5% DMSO	15			
7.5% DMSO + 10μM ROCK inhibitor	15			20
7.5% DMSO + 2.5%PEG	15		5	
7.5% DMSO + 2.5%PEG + 10 μM ROCK inhibitor	15		5	20
10% PROH		20		
7.5% PROH + 2.5%PEG		15	5	

### Assessment of cell viability and cell recovery after freezing

Cell viability immediately after cryopreservation at different conditions was detected using a Propidium Iodide (PI) (BD Bioscience, UK) staining method, which detects integration of cell membrane indicating dead cells. The cells were stained with 1 μM of PI for 1 min in dark after CPA was removed. In each measurement, twenty thousand cell events were measured. The results were expressed as the percentage of living cells.

The cells, cryopreserved using 10 % DMSO, after thaw and DMSO removal were plated in the absence of inhibitor was used as the freezing control condition.

The frozen cells, cryopreserved by 10% DMSO, 10% DMSO + 10 μM ROCK inhibitor, 7.5% DMSO, 7.5% DMSO +10 μM ROCK inhibitor, 7.5% DMSO +2.5% PEG and 7.5% DMSO +2.5% PEG+10 μM ROCK inhibitor, after thaw and CPA removal were resuspended in the culture medium and then were plated at 1 x 10^5^ cells/well (48-well plate) on matrigel-coated plates for feeder-independent culture or on a feeder layer for feeder-dependent culture in the presence of 10 μM Y-27632, or in the presence of 10 μM Y-27632 and 1μM pifithrin-μ, or in the absence of inhibitor at the first day of culture. After 4 days of culture, the cells from feeder-independent culture were dissociated, re-suspended and counted. After 5 days of culture the cells from feeder-dependent culture were stained with 5-bromo-4-chloro-3-indolyl phosphate and nitro blue tetrazolium (BCIP-NBT) (Sigma-Aldrich, UK), and were incubated at room temperature in the dark. Images were taken using a Nikon Coolscope.

### Assessment of apoptosis and caspase activity

Frozen cells from feeder-independent, cryopreserved by 10% DMSO, 7.5% DMSO +2.5% PEG, 10% DMSO + 10 μM ROCK inhibitor and 7.5% DMSO +2.5% PEG+10 μM ROCK inhibitor, after thaw and CPA removal were plated in the absence of inhibitor [R(-)P53(-)]. The cells cryopreserved by 10% DMSO was further plated in the presence of 10 μM Y-27632 [R(+)P53(-)], and in the presence of 10 μM Y-27632 and 1 μM pifithrin-μ [R(+)P53(+)]. For assessment of apoptosis, after 2 h of plating, the cells were stained with the combination of Annexin V(A)-FITC and PI(P) (BD Bioscience, UK), which can identify the cells at an earlier stage of apoptosis [A(+)P(-)] or at late stage of apoptosis [A(+) P(+)].^20^ A-P, incubated at room temperature for 15 min in the dark, and kept on the ice before analyze using flow cytometry. The results were expressed as the percentage of experiencing apoptosis. For caspase activity assay, the cells after regular passage and the cells cryopreserved by 10% DMSO and 7.5% DMSO +2.5% PEG after thawing, and CPA removal were resuspended and plated at the density of 7.5 x 10^4^ cells/well (96-well plate) at the condition of R(-)P53(-), R(+)P53(-), or R(+)P53(+). After 2 h and 1 day of plating, an equal volume of reagent, Caspas-Glo 8 and Caspase-Glo assay (Promega, USA), was added to the tested well for 1 h in the dark at room temperature. The caspase-8 and caspase-9 activity was read by a luminometer (Lucy, UK).

### Assessment of intracellular reactive oxygen species and F-actin

The cells from regular passage were dissociated, and the cells from cryopreservation were thawed,and CPAs were removed. To assess ROS, the cells were stained with dihydroethidine (DHE) (Sigma-Aldrich, UK), which is fluorescence sensitive at an excitation wavelength of 488 nm and emission wavelength of 567 nm once it is oxidized, for superoxide anion generation for 30 min on ice in the dark. To quantify F-actin, the cells were fixed in PBS containing 4% formaldehyde (Sigma-Aldrich, UK) for 15 min. The cells were pelleted by centrifuge and resuspended in PBS supplemented with 0.1% Triton (Sigma-Aldrich, UK) and 0.33 μM rhodamine phalloidin (Invitrogen RIBCO, UK) for 15 min at 4°C.^21^ F-actin content was quantitatively determined by flow cytometry. Twenty thousand cells were measured at each condition. ROS and f-actin were determined by flow cytometry. The results were expressed as DHE or f-actin fluorescence intensity.

### Immunocytochemical characterisation

Immunocytochemical characterisation was determined for the colonies from feeder-independent and feeder-dependent culture at passage twoafter cryopreservation. Immunostaining was performed by using a Human Embryonic Stem Cell Marker kit (R&D Systems, USA). For intracellular staining of Oct4 and Nanog, the cells after 6 days of culture were washed with PBS, and then fixed with 4% formaldehyde in PBS for 20 min at room temperature, permeabilised and blocked with 0.1% Triton X-100 (Sigma-Aldrich, UK), 1% Bovine Serum Albumin (BSA, Sigma-Aldrich, UK) and 10% normal donkey serum (Jackson ImmunoResearch, USA) in PBS for 45 min at room temperature. With surface marker, SSEA4, no permeabilisation was required. The cells were blocked with 1% BSA and 10% normal donkey serum for 45 min at room temperature. Primary antibodies, goat anti-Nanog polyclonal antibody, goat anti-Oct4 polyclonal antibody, and mouse anti-SSEA4 monoclonal antibody, were added to the cells at a final concentration of 10 μg/mL in PBS at 4°C overnight, washed twice with 1% BSA in PBS to remove unbounded primary antibody. Secondary antibodies, FITC-conjugated donkey anti-goat IgG and FITC-conjugated donkey anti-mouse IgG (all from Jackson ImmunoResearch, USA) were diluted at 1:100 in PBS containing 1% BSA. The diluted secondary antibody was applied to the cells for 60 min at room temperature, washed with PBS for three times. The slides were mounted with the SlowFade kit containing DAPI (Invitrogen GIBCO, UK). Stained cells were visualized by fluorescence microscopy (Nikon, Japan) connected to a cooled charge-coupled device video. Images were collected with ART-1 software.

### Statistics analysis

At least three separate experiments were conducted for each tested condition. Data are presented as means ± SD of the means for the experiments. The statistical significance was assessed using one-way ANOVA. A probability of *P* < 0.05 was considered significant.

## Results

### Cell viability and recovery after cryopreservation

**Figure 1:**
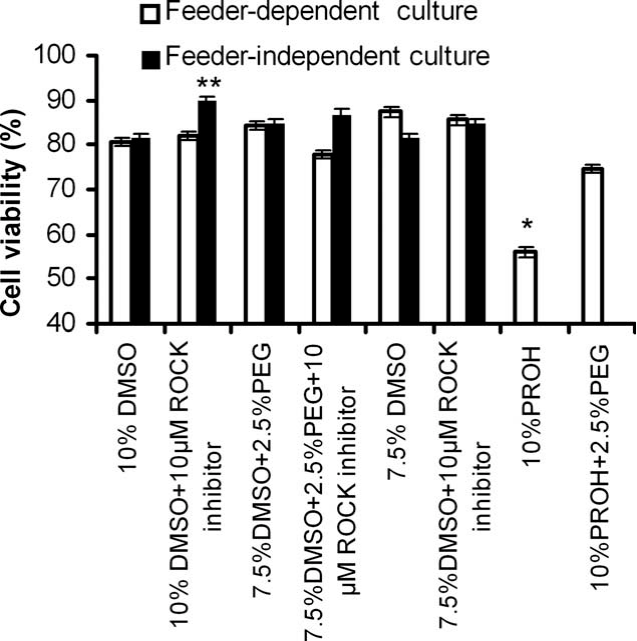
Cell viability immediately after thawing Results are expressed as the mean of independent experiments ± the standard deviation (*n* = 3), Statistical analysis was performed using one-way ANOVA. *P < 0.05, compared with the results obtained in 10% DMSO.

The percentage of living cells in the whole cell population is shown in Figure 1. For both types of culture no significant difference in cell viability was observed, except for using 10% PROH as freezing solution (*P* = 0.012, *n* = 3). The presence of ROCK inhibitor in the freezing solutions did not enhance the cell viability on any of freezing conditions, except for 10 % DMSO (P = 0.03056, *n* = 3).

**Figure 2:**
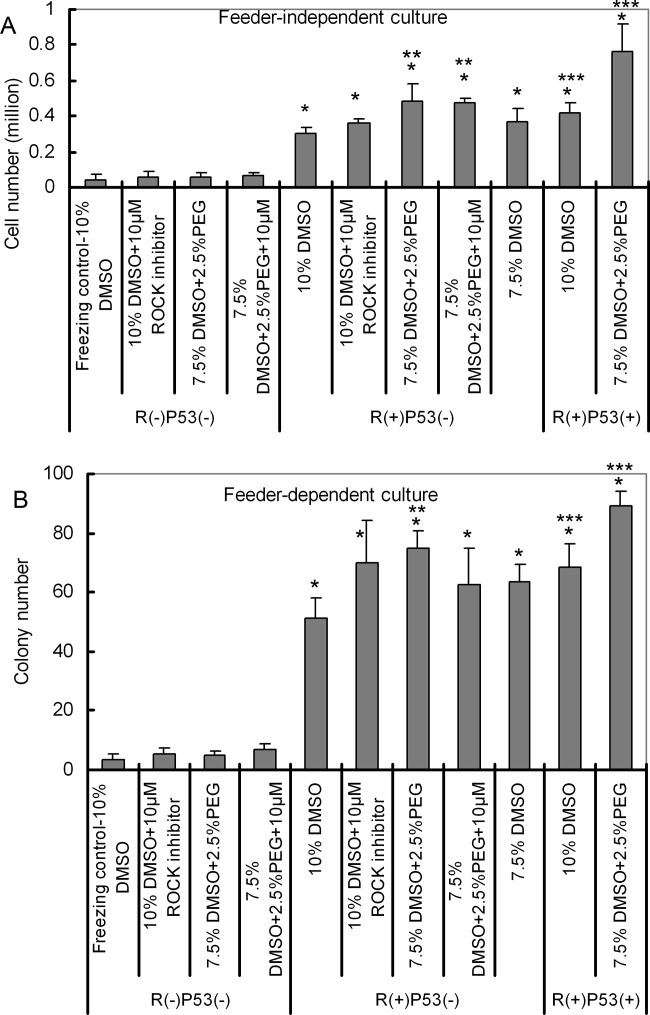
Cell growth after cryopreservation for feeder-independent and feeder-dependent culture A: For feeder-independent culture. B: For feeder-dependent culture. R(+)/R(−): with or without Rock inhibitor; P53(+)/P53(−): with/without p53 inhibitor in the subsequent culture medium. Results are expressed as the mean of 3–4 independent experiments ± the standard deviation. Statistical analysis was performed using one-way ANOVA, *P < 0.05. Compared with the results obtained in 10% DMSO. **P<0.05, compared with the results between 10% DMSO in the group of R(+)P53(−) PEG, ***P < 0.05, compared with the results between R(+)p53(−) and R(+)p53(+).

Cell growth after cryopreservation is used as an indicator to show how efficient the protocol of cryopreservation of dissociated hES cells. We first determined the effects of PEG and DMSO concentration in the freezing solution on cell recovery. As shown in Figure 2, the presence of PEG in the freezing solution significantly increased the cell recovery compared with 10% DMSO for both culture systems. Nevertheless, lower DMSO concentration led to improvement in cell recovery, not significant.

Then the effect of p53 inhibitor in the subsequent culture medium on cell recovery was investigated. After cryopreservation, the presence of p53 inhibitor during subsequent culture produced a great effect on cell recovery for 10% DMSO and 7.5% DMSO +2.5% PEG in both culture systems (Figure 2).

Finally, the role of Y-27632 treatment during cryopreservation process was determined. Cell recovery was not increased by the presence of Y-27632 in the freezing medium for freezing conditions (10% DMSO and 7.5% DMSO +2.5% PEG) in all culture systems (Figure 2).

### Apoptosis rate and caspase activty

**Figure 3:**
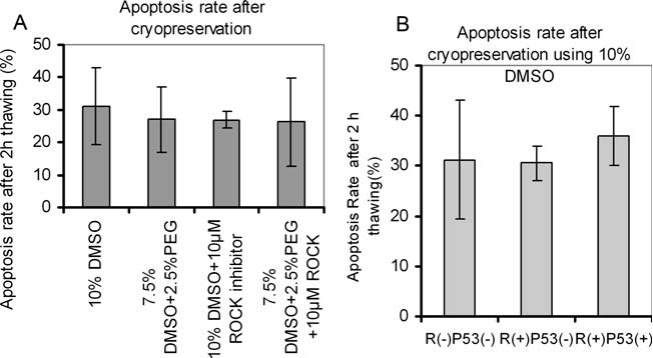
Apoptosis rate after 2 h plating for feeder-independent culture A: Apoptosis rate for the cells frozen at the different conditions, B: Apoptosis rate for the cells frozen by 10% DMSO and then cultured at different conditions after thawing, R(+)/R(−): with or without Rock inhibitor. P53(+)/P53(−): with or without pifthrin μ inhibitior. Results are expressed as the mean of independent experiments ± the standard derivation (*n* ≥ 3). Statistical analysis was performed using one-way ANOVA.

We first examined the effect of the composition of freezing solutions on cell apoptosis rate. Figure 3A shows no significant difference in apoptosis rate after 2 h re-plating was observed for all test conditions. Then we looked at whether the presence of ROCK inhibitor and pifithrin-μ in the subsequent culture medium could affect the cell apoptosis rate after cryopreservation. The cells cryopreserved by 10% DMSO were thawed and re-plated at the condition of R(-)P53(-), R(+)P53(-), or R(+)P53(+). As shown in Figure 3B, around 30% of cells experienced early apoptosis for all tested conditions.

**Figure 4:**
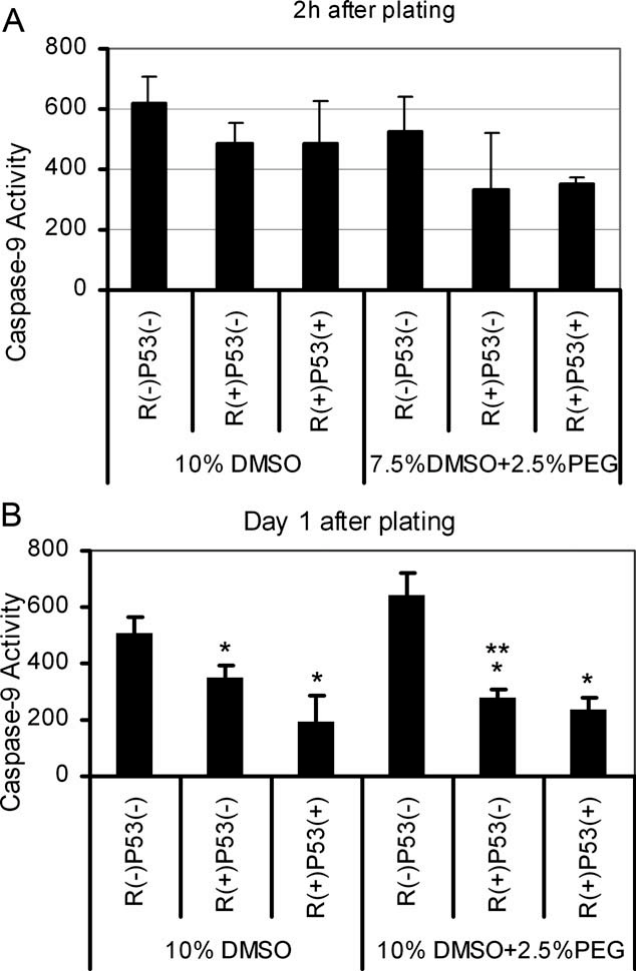
Caspase-9 activity after 2 h and 1 day of plating after cryopreservation for feeder-independent culture A: Caspase-9 activity after 2 h of plating, B: Caspase-9 activity after 1 day of plating. Rock inhibitor present/absent R(+)/R(−) at the first day of culture after cryopreservation, pifithrin-μ present/absent P53(+)/P53(−) at the first day of culture after cryopreservation. Results are expressed as the mean of 3–4 independent experiments ± the standard deviation. Statistical analysis was performed using one-way ANOVA. *P < 0.05, compared with the results in R(−)p53(−) for 10% DMSO and 7.5% DMSO +2.5% PEG groups. **P < 0.05, compared with the results in R(+)p53(−) for 10% DMSO and 7.5% DMSO +2.5% PEG groups.

Caspase activity was used to indicate the apoptosis of the cells after cryopreservation. The cells after cryopreservation using 10% DMSO and 7.5% DMSO +2.5% PEG were cultured at the condition of R(-)P(-), R(+)P(-) or R(+)P(+). Caspase-9 was measured after 2 h and 1 day plating of frozen-thaw cells for feeder-independent culture, as shown in Figure 4. After 2 h of plating, no significant difference was observed between different freezing groups at the corresponding culture conditions. At day 1, around 30% lower was obtained by the presence of ROCK inhibitor (*P* = 0.003 and *P* = 0.0003 for 10% DMSO and 7.5% DMSO +2.5% PEG, *n* = 4, respectively). A further reduction was achieved in the presence of p53 inhibitor and Y-27632 (*P* = 0.025, *n* = 3) when 10 % DMSO was used. However, when 7.5% DMSO +2.5% PEG was used, the presence of p53 inhibitor did not result in a significant decrease in caspase-9 activity.

### Assessment of ROS and F-actin

**Figure 5:**
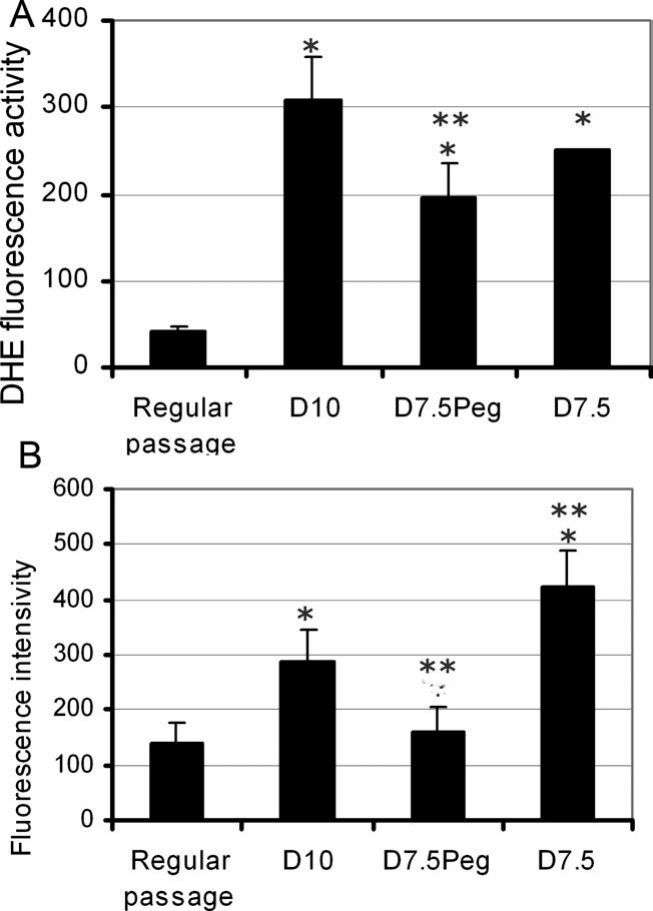
Intracellular superoxide anion and the amount of F-actin from feeder-independent culture before and after cryopreservation at different freezing conditions A: Intracellular superoxide anion. B: The amount of F-actin. Results are expressed as the mean of 3–4 independent experiments ± the standard derivation. Statistical analysis was performed using one-way ANOVA, *P < 0.05, compared with the results from regular passage. **P < 0.05 compared with the results from 10% DMSO. D10: 10% DMSO, D7.5Peg: 7.5% DMSO +2.5% PEG, D7.5: 7.5% DMSO.

Superoxide was evaluated by measurement of DHE intensity for regularly passaged cells, and the cells cryopreserved by 10% DMSO, 7.5% DMSO +2.5% PEG and 7.5% DMSO, as shown in Figure 5A. We noticed that the superoxide generation was dramatically increased by cryopreservation compared with regular passage. In comparison with 10% DMSO, the level of superoxide anion generation was significant reduced by using 7.5% DMSO +2.5% PEG.

We compared the amount of F-actin in the cells from feeder-independent culture after regular passage and after cryopreseration using 10% DMSO, 7.5% DMSO +2.5% PEG and 7.5% DMSO. The amount of F-actin in the cells is shown in Figure 5B. Compared with regular passage, a significant increase in F-actin occurred after cryopreservation using 10% DMSO and 7.5% DMSO. For 7.5% DMSO +2.5% PEG, however, the change in F-actin content was not significant. Moreover, F-actin content in the cells cryopreserved by 7.5% DMSO +2.5% PEG was much lower than that in the cells cryopreserved by 10% DMSO and 7.5% DMSO.

### Immunocytochemical characterisation

**Figure 6:**
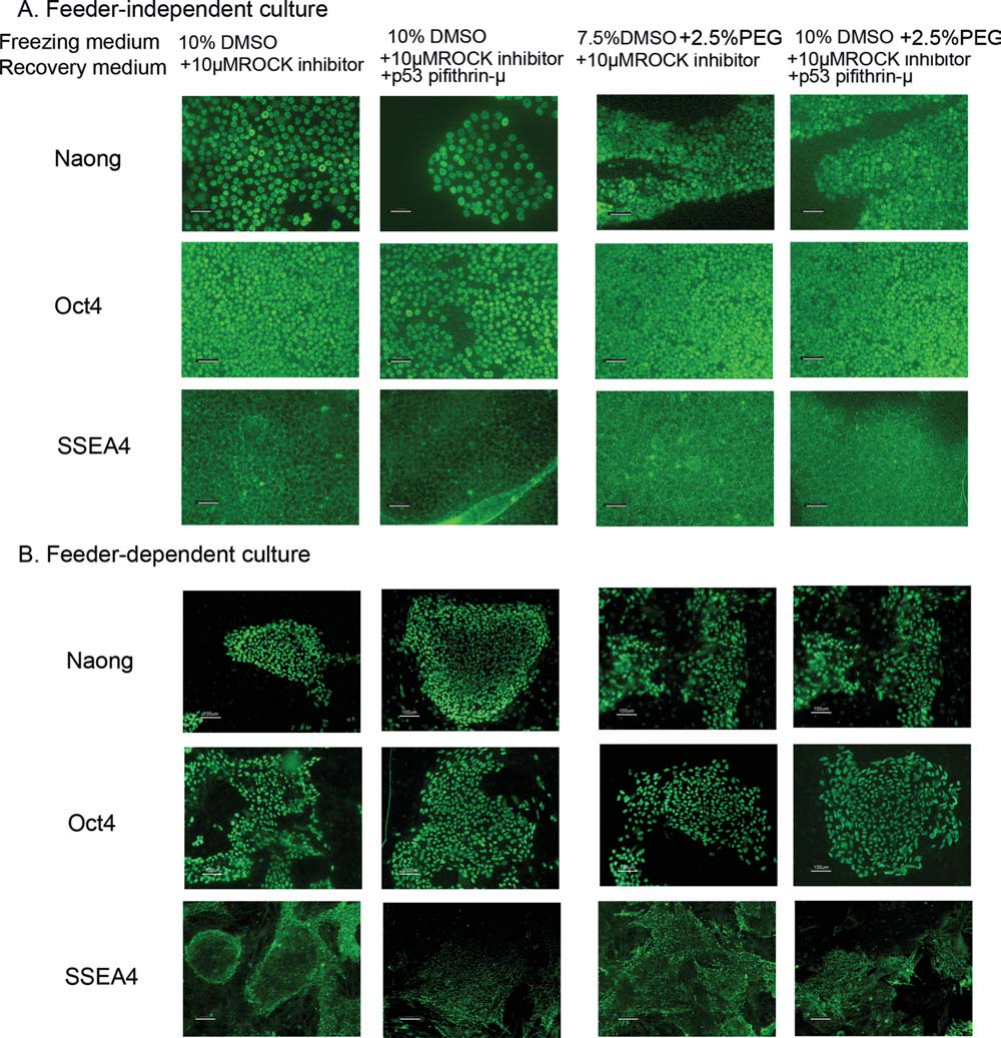
Expression of undifferendiated marker, nuclear markers, OCT4 and Nanog, and surface marker, SSEA4, for hES cells at passage two after cryopreservation using 10% DMSO and 7.5% DMSO +2.5% PEG The cells after cryopreservation were cultured in the presence of ROCK inhibitor, or in the presence of ROCK inhibitor and pifithrin-μ. A: Feeder-independent culture, B: Feeder-dependent culture. Scale bar: 50 μm for feeder-dependent culture and 25 μm fro feeder-independent culture, respectively.

Typical images for assessment of the undifferentiated status of hES cells after cryopreservation using 10% DMSO and 7.5% DMSO +2.5% PEG, and cultured in the presence of ROCK inhibitor or in the presence of ROCK inhibitor with pifithrin-μ at the first day of culture after thawing are shown in Figure 6, and negative control is shown in supplementary Figure 1. The presence of PEG in the freezing solution did not affect the undifferentiated stage of the cells. Moreover, the cells from both culture systems also maintained their undifferentiated state when pifithrin-μ was present at the first day of culture after thawing.

## Discussion

The conventional slow-freezing and fast-thawing protocol using 10% of DMSO as a cryoprotectant has not been successful for cryopreservation of hES cells yet. The obstacle we face in this method is an extremely low cell survival rate, around 10%^8^. The presence of Y-27632 at day 1 of culture during post-thawing culture can overcome this low recovery rate and significantly enhances the cell survival rate,^10^, ^11^ but can be further improved. In our study, an efficient protocol for cryopreservation of hES cells and subsequent culture was developed. The introduction of PEG into 7.5% of DMSO freezing solution incorporating Y-27362 in the subsequent culture results in higher cell recovery rate than the conventional method. In addition, the combination of Y-27632 and pifithrin-μ in the subsequent culture allows a further increase in the cell recovery rate. With this novel protocol for cryopreservation and subsequent culture, the cells retain an undifferentiated status. This novel protocol provides a convenient and efficient method for cryopreservation of a large quantity of hES cells.

To identify hES cell behaviour during cryopreservation and subsequent culture, a pure population of hES cells is required. Hence, to avoid the interference caused by the existence of feeder cells, we used feeder-independent culture to investigate the change in caspase-9 activity, intracellular ROS level and F-actin content during cryopreservation and subsequent culture.

The cell survival rate after cryopreservation is regarded as an indicator whether or not the freezing protocol is efficient. Our results showed that around 80% of cells were alive immediately after thawing for both culture systems at all tested conditions except for 10% PROH. This implies that low cell recovery after slow-freezing cryopreservation is associated with apoptosis rather than necrosis induced by cryoinjury.^8^ In addition, as no colony was formed after cryopreserved by 10% PROH +2.5% PEG and 10% PROH (data not shown) consistent with previous study,^22^ we did not carry out further experiments on these freezing conditions.

Therefore, inhibition of apoptosis during cryopreservation is thought to be a solution to increase the cell recovery rate. When 10% DMSO is used, ROCK inhibitor, Y-27632, is demonstrated to inhibit apoptosis in regular culture of hES cells.^9^ We further demonstrated that as in the previous studies,^10^, ^11^ the presence of Y-27632 in the subsequent culture medium led to a significant increase in cell colony formation. We also confirmed that the addition of ROCK inhibitor in the freezing solution, 10% DMSO and 7.5% DMSO +2.5 % PEG, but not in the subsequent culture medium, did not help to improve the cell viability (Figure 1) and cell recovery (Figure 2) which is inconsistent with previous findings.^10^ This inconsistency in the role of Y-27632 presented in the freezing medium on recovery could be caused by the differences in cell lines and culture system used.

However, as a specific inhibitor of the ROCK family, the role of Y-27632 in apoptosis is not clear yet.^23^ Since the apoptosis rate was not affected by the presence of Y-27632 in the subsequent culture medium 2 h after plating, we further measured caspase-9 activity after 2h and 1 day of plating following cryopreservation for the group of 10% DMSO and 7.5% DMSO +2.5% PEG. At day 1, we found that caspase-9 activity was inhibited by the presence of ROCK inhibitor which is consistent with the previous study.^9^

ROS production is regarded as one of factors to cause damage induced by cryopreservation, and leads to cellular damage which cannot be recovered. To find out whether the improvement in cell recovery was caused by the presence of PEG which can provide protection against damage by decreasing ROS elevation,^15^ or by the decrease in DMSO concentration, we evaluated cell recovery and ROS production by the cells cryopreserved by 7.5% DMSO. It is certain that 7.5% DMSO is not superior to 10% DMSO (Figures 2 and 5A). In 7.5 % DMSO +2.5% PEG, the low concentration of DMSO having low toxicity to the cells and the presence of PEG both contribute to the improvement of cell recovery. Hence, we concluded that 7.5% DMSO with 2.5% PEG is more efficient for cryopreservation of dissociated hES cells than the conventional method.

Regulation of actin assembly and disassembly linking to an external stimulus is essential for cellular processes such as cell motility, apoptosis, cell shape, and plasma-membrane integrity.^24^ We noticed that F-actin content inside hES cells was increased dramatically after cryopreservation using 10% DMSO and 7.5% DMOS, compared with regular passage. A total of 7.5% DMSO +2.5% PEG did not eliminate the increase in F-actin, however, it alleviated the change in F-actin after cryopreservation (Figure 5B). This mediation could be associated with reduction in caspase-9 activity as well. The alleviation in F-actin content could contribute to the protection provided by PEG against damage caused by cryopreservation.

In addition, the tumour suppressor, p53 has an important role in regulation of various cellular processes such as apoptosis, differentiation, and genomic integrity.^25^ We demonstrated that when 10% DMSO or 7.5% DMSO +2.5% PEG was used for cryopreservation of hES cells, the combination of Y-27632, and p53 inhibitor in the subsequent culture medium significantly enhanced the cell recovery compared with Y-27632 alone. However, the cell recovery rate could not be improved by the presence of p53 inhibitor alone (data not shown). It is interesting to note that the presence of p53 inhibitor in the subsequent culture medium led to reduction in caspase-9 activity for both freezing conditions (Figure 4). This reduction contributes to improve the cell recovery consistent with the previous report.^21^

Maintenance of undifferentiated status is a major challenge in cryopreservation of hES cells.^26^, ^27^ At passage 2 after cryopreservation, all colonies expressed the nucleus marker, Oct4 and Nanog, and surface marker, SSEA4, as the previous report.^10^, ^11^ Further quantitative study is needed to be utilized to clarify the effect of PEG and p53 on cell pluripotency. In addition, epigenetic study is also needed to confirm that the hESCs are still karyotipically normal after cryopreservation using PEG and recovery using p53.

In summary, we have developed an efficient method for cryopreservation and subsequent culture of dissociated hES cells. This method can be easily scale-up for mass cryopreservation of hES cells to satisfy the widespread applications of hES cells in clinical area.
